# Association between experience of specific side-effects and contraceptive switching and discontinuation in Uganda: results from a longitudinal study

**DOI:** 10.1186/s12978-021-01287-5

**Published:** 2021-11-27

**Authors:** Linnea A. Zimmerman, Dana O. Sarnak, Celia Karp, Shannon N. Wood, Saifuddin Ahmed, Fredrick Makumbi, Simon P. S. Kibira

**Affiliations:** 1 Department of Population, Family and Reproductive Health, Johns Hopkins Bloomberg School of Public Health, Baltimore, USA; 2 Department of Epidemiology and Biostatistics, School of Public Health, Makerere University, Kampala, Uganda; 3 Department of Community Health and Behavioural Sciences, School of Public Health, Makerere University, Kampala, Uganda

**Keywords:** Contraceptive side-effects, Contraceptive continuation, Contraceptive switching, Longitudinal, Menstrual bleeding, Sexual experience, Uganda

## Abstract

**Background:**

There is substantial evidence that contraceptive side-effects are a major deterrent to consistent use of contraception but few studies in low- or middle-income countries explore the role of specific side-effects on contraceptive use dynamics. This study used population-based, longitudinal data to explore the effect of specific side-effects on contraceptive continuation, discontinuation, and switching in Uganda.

**Methods:**

Data for this study come from two rounds of survey data collection in Uganda: PMA2020’s sixth cross-sectional survey and a follow-up survey conducted 1 year later. The main outcomes of interest were discontinuation and switching among users of hormonal contraceptive methods (implants, injectables and oral pill) and the IUD at baseline (n = 560). Multivariable logistic regressions assessed the association of experiencing specific side-effects (more bleeding, less bleeding, irregular bleeding, increased dryness/reduced libido, and physical discomfort) with discontinuation and switching 1 year later, adjusting for socio-demographic characteristics, type of method, and length of use. We also examined the differential effects of side-effects between discontinuation and switching risks.

**Results:**

About 23% of hormonal and IUD contraceptive users reported experiencing side-effects at baseline survey. Overall, discontinuation and switching were higher among injectables and pill users, compared to IUD and implants users. Reporting more bleeding or less bleeding increased the odds of discontinuation and switching by 2.74 (95% CI 1.00–7.51) and 1.86 (1.04–3.34), respectively. There was no significant difference in discontinuation and switching by side-effects.

**Conclusions:**

Greater attention should be paid to understanding the unique contributions of side-effects to contraceptive behavior using population-based data. While about a quarter of women reported experiencing side effects, those who experienced bleeding specific side effects were at higher risk of contraceptive discontinuation and switching. Providing greater individualized care that includes information and counseling about common side-effects, how they may impact daily life, and how tolerable these effects may be is necessary.

## Plain English summary

Research has shown that experiencing side-effects is related to stopping use of contraception, even when women wish to avoid pregnancy. Most research, however, does not differentiate between distinct side-effects, such as increased bleeding or changes to sexual experience, and instead combined all into “side-effects or health concerns”. We used data from 560 women in Uganda, who were interviewed twice, 1 year apart, to see if women who reported different side-effects at the first interview were more likely to stop using contraception or switch to a different contraceptive method than women who did not report experiencing side-effects. We found that increased or decreased menstrual bleeding was associated with a higher odds of contraceptive discontinuation and switching, Contraceptive discontinuation or switching was not different by women’s reporting of vaginal dryness/reduced libido or physical discomfort, such as cramping. It is important to understand what side-effects are likely to motivate stopping or switching contraception so that education and counseling can inform women of side-effects they may experience, help them choose the best method based on what side-effects they deem important, and if necessary, aid in switching contraceptive methods.

## Background

There is substantial evidence that contraceptive side-effects, either experienced or perceived, are a major deterrent to consistent use of contraception [1–4]. Across diverse settings, women report experiencing changes to menstrual bleeding, abdominal pain, weight gain, changes to sexual experience, and non-specific “weakness” as influencing their decision-making about contraception [5–12]. Though providing critical contextual data, qualitative data are unable to assess how widespread experiences are, how these experiences contribute to contraceptive use dynamics at a population level, and what side-effects may be largely tolerated by women versus those that lead to discontinuation or switching.

Population-based national surveys with publicly available data on family planning, such as the Demographic and Health Survey (DHS), Performance Monitoring and Accountability (PMA2020, now Performance Monitoring for Action (PMA)) and the Multiple Indicator Cluster Survey (MICS), do not include questions that identify specific side-effects, instead identifying only generalized “side-effects” or “health concerns” as a reason for contraceptive discontinuation [8, 13, 14]. Recent efforts have called for increased specificity of side effect data. Specifically, one review indicates that while changes to menstrual bleeding, including increased bleeding, amenorrhea, and irregular bleeding and spotting, have been shown to be particularly influential on contraceptive use, there is a lack of nationally representative data available on the experience of these side-effects [15]. Another recent review of contraceptive-induced changes to sexual experience similarly noted both the importance that is placed on these changes by women and the critical dearth of research within low- and middle-income countries (LMICs) [16]. Similar knowledge gaps exist around the prevalence and consequences of additional side-effects, such as nausea and headaches [5, 12, 17]. This lack of knowledge limits our understanding of both the prevalence and effects of specific contraceptive induced side-effects on contraceptive behaviors.

Beyond understanding the prevalence of side-effects in a population, it is also important to understand what factors may influence the tolerability of side-effects; that is, what factors are associated with women continuing to use a method in the presence of side-effects. This question is particularly relevant to consider in the context of LMICs, as the vast majority of research has been limited to high-income settings or conducted as part of clinical trials [18–23]. Tolerability of specific side-effects may differ for a variety of social, cultural, and economic reasons; how side-effects are interpreted, treated (e.g. availability and accessibility to menstrual hygiene products or treatment for cramping), and the economic and physical consequences when they are experienced are heavily influenced by context and quality of available services [6, 10, 24–26]. Findings from high-income countries should not, therefore, be applied to LMIC contexts nor should findings from one LMIC be rotely applied to another. Gaining a more nuanced understanding of the characteristics of women in specific settings who tolerate side-effects and continue using contraception, versus those that discontinue, may thus provide valuable lessons to program managers and health providers.

Finally, limited research has explored how the experience of specific side-effects may motivate switching to a different method versus discontinuing contraception altogether. Contraceptive switching has significant programmatic implications for (1) promoting the use of more-effective, long-acting methods from lesser effective methods; (2) addressing discontinuation related to experienced side-effects by promoting alternative contraceptive methods; (3) ensuring that women are able to choose a method that best aligns with their reproductive goals—a critical component of high-quality, rights-based family planning programs [27–30]. While the outcome of switching is generally considered positive due to sustained protection against unintended pregnancy, there is significantly less known about what motivates switching versus discontinuation and what facilitates or impedes this decision. To our knowledge, the only prospective study in LMICs to assess specific side-effects and their effect on contraceptive switching versus discontinuation was conducted by Barden-O’Fallon and colleagues among women in Honduras [31]. The authors found that Honduran women who experienced amenorrhea or heavy menstrual bleeding were significantly more likely to discontinue than switch methods and that urban women were significantly more likely to switch methods than rural women, underscoring the simultaneous roles of side-effects and sociodemographic characteristics on women’s contraceptive practices. However, the study did not compare switching and discontinuing separately to assess the effect of experiencing side-effects on these behaviors, relative to continuation. Despite the logical contribution of side-effects to women’s decisions to adopt a different method or stop using contraception entirely, there is a clear dearth of research in this area.

To better understand the prevalence of different side-effects and their association with contraceptive use dynamics, a longitudinal study was conducted in Uganda. This study aimed to estimate the prevalence of a range of specific side-effects that were experienced among users of hormonal contraception and the IUD and to establish the association between these side-effects and contraceptive discontinuation, switching, or continuation 1 year later. Additionally, we sought to identify relevant socio-demographic characteristics associated with discontinuation and with switching.

## Methods

### Study setting

Uganda had a population of about 44.3 million in 2019, with nearly half its population under age 15 [32]. Uganda is characterized by high fertility; the total fertility rate (TFR) in 2016 was 5.4 births, down from 6.9 in 2000–2001 [13]. Modern contraceptive use in Uganda has also increased in recent years, from 21% in 2014 to 30% in 2018. Injectables were the most common method used in 2018 (39%), followed by implants (20%) (ibid). Despite the increase in contraceptive use, high rates of contraceptive discontinuation persist; nationally, 20% of IUD users, 17% of implant users, 36% of injectable users, and 46% of pill users discontinued in the 1st year of use, while in need of protection against unintended pregnancy (ibid).

### Study overview and sampling

Data for this study come from two rounds of PMA2020 data collection in Uganda. Specifically, in PMA2020 Uganda’s sixth cross-sectional survey [14], conducted from April to June 2018 (hereafter referred to as baseline), the consent and design were modified to enroll women in a longitudinal study. A follow-up survey was conducted approximately after 1-year from May to June 2019.

PMA2020 is a multi-stage cluster, nationally representative survey of women age 15–49. In the baseline survey, 110 Enumeration Areas (EA) were selected using probability proportional to size sampling and all occupied households were enumerated. Forty-four households were randomly selected within each EA, consented, and interviewed. All women age 15–49 who were either usual members of the household or who slept in the household the night before were approached for interview, and if consented, interviewed by a trained interviewer. A total sample of 4288 women were interviewed in the baseline survey (response rate: 96.9%); the majority (95.5%; n = 4095) of women agreed to participate in the follow-up survey. Further information on the design of PMA2020 surveys is available from www.pmadata.org and Zimmerman et al. [33].

At follow-up, interviewers returned to the households of women who completed the baseline survey and re-consented women to participate in the follow-up survey. We were able to relocate and successfully interview 2755 women from the original sample, resulting in a follow-up rate of 67%. Due to potential bias from loss to follow-up, we constructed an inverse probability weight from estimated propensity scores to adjust differential loss to follow-up. Using the total sample of women from baseline (n = 4288), we adjusted for the probability of being interviewed at follow-up accounting for age (5-year age groups), education (none, primary, secondary and higher), marital status (currently married/in-union, not married), wealth (five quintiles), and residence (urban,rural). The original baseline individual female weight was then multipled by the inverse probability weight to construct the final weight in the analyses. We assessed differences between women who were in the analytic sample (outlined further below) and followed-up (n = 560) versus those that met the criteria for the analytic sample but were lost to follow-up (n = 233). Overall, women who were lost-to-follow-up were younger, less likely to be married, more likely to live in an urban area and be of low parity, less likely to say they did not want to have any more children, and more likely to have used their method fewer than 12 months. Overall, their contraceptive related outcomes (method choice and experience of side-effects) were similar to the study sample (shown in Appendix Table 6).

### Analytic sample

**Fig. 1:**
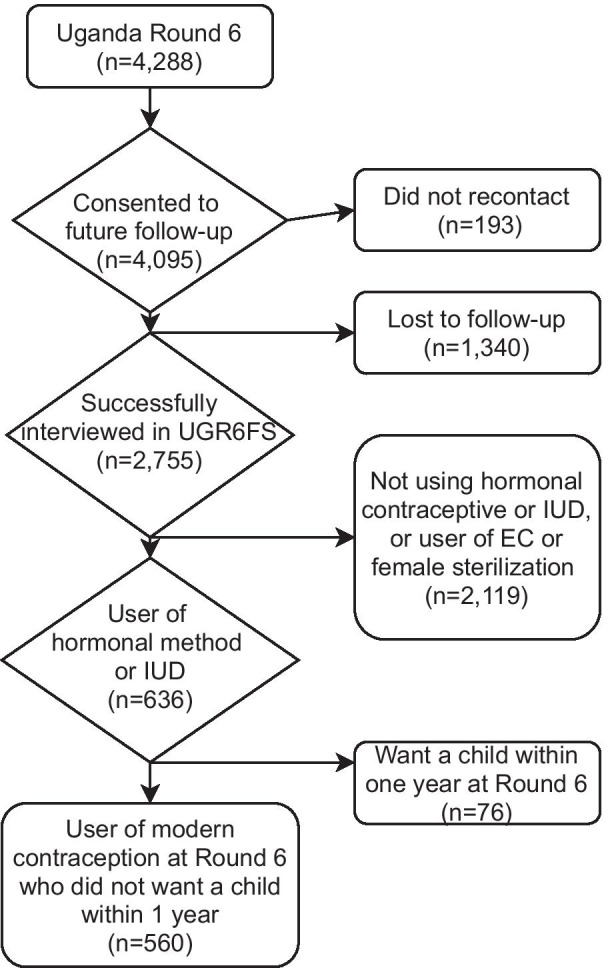
Selection process for analytic sample

This analysis was restricted to women who reported currently using hormonal contraception (implant, injectable, pill) or the IUD at baseline. Of note, we are unable to differentiate between hormonal and non-hormonal IUD use. Users of emergency contraception were excluded due to the periodicity in its use, and female sterilization users were excluded as they would not be able to discontinue or switch methods. Condoms and other barrier and traditional methods were excluded as systemic side-effects were not anticipated from using these methods. Women who wanted to have another child within 1 year at baseline were excluded (n = 76) as they may have discontinued to become pregnant irrespective of their side-effects experience. In total, 560 women were included in this analysis; the flowchart of sample selection process is shown in Fig. 1. Data were complete for all observations used in analysis. The follow-up rate for the analytic sample was 71%; 560 users were relocated out of 793 pill, implant, injectable, and IUD users at baseline who did not want to become pregnant in the next year.

### Measures

At baseline, women were asked to report whether they were currently experiencing any side-effects, and if so, to specify all that they were currently experiencing (with multiple-response options; responses were not prompted or read aloud). The list of side-effects was drawn from the literature on clinically documented side-effects, and those that have been reported in qualitative research in Uganda. The list was reviewed with the in-country team and pilot tested; the complete list of side-effects is shown in Appendix Table 7. Women were coded as 1 if they reported experiencing each side-effect and 0 if not.

The outcomes related to contraceptive behavior were defined based on the woman’s contraceptive use status at the time of follow-up survey. Specifically, women were categorized in to three groups: either continued (using the same method at baseline and follow-up), switched (using a different method at baseline and follow-up), or discontinued (not using a method at follow-up). First, we examined the odds of method-specific discontinuation, which included discontinuation and switching, compared to continuation, and then we examined discontinuation compared to switching by side-effects reporting. We assessed the distribution of each outcome by method type (implant, IUD, injectables, and oral pills).

### Covariates

All analyses were adjusted for the following socio-demographic variables measured at baseline: age (included as a continuous variable), marital status (binary variable indicating married/in-union), residence (urban or rural), education (categorical variable indicating none, primary, or secondary and above), and parity (categorical variable of 0–1, 2–3, or 4+ children). We combined nulliparous and primiparous women, as there were only nine nulliparous women included in the analytic sample. Due to low-prevalence of side-effects and sample size limitations, we created a binary variable for wealth, indicating whether the respondent resided in a household in one of the lowest two wealth quintiles (0) or the wealthier three (1). We adjusted for method used at baseline by including a categorical variable of method type. To account for the fact that side-effects may be more frequent when initiating a method and resolve over time, we included a binary variable indicating whether the woman started her method less than 12 months prior to the baseline interview. We used a 12-month time frame to align with previous research [31]. Finally, we adjusted for stated fertility intentions at baseline (wanted child within 2 years, wanted child after 2 years, wanted no more children).

### Analysis

Exploratory analyses assessed the prevalence of each side-effect for further modeling considerations. Based on sample size, the following side-effects were combined: “uterine cramping”, “cramping”, “headache”, “nausea”, and “weakness” (“physical discomfort”); “vaginal dryness” and “lowered libido” (“vaginal dryness/lowered libido”); “spotting” and “irregular bleeding” (“irregular bleeding”).

We fit two multivariable logistic regression models: first, we assessed the risk of discontinuing or switching, relative to continuing, among women who reported experiencing the side-effect compared to women who did not, and then examined the the risk of discontinuation compared to switching after excluding the women who continued. As women could experience multiple side-effects simultaneously, we ran adjusted models that included all side-effects and the covariates listed above. Sensitivity analyses were conducted with the inclusion of each side-effect independently and results were largely consistent (results not shown).

Descriptive analyses (Tables 1, 2 and 3) applied the survey weights described above. Due to small sample sizes and because weighting increases variances and design-effects substantially, we tested the efficiency of the application of weights in the logistic regressions and to maximize efficiency, we followed guidance from Korn and Graubard and did not apply the survey weights to the regression models [34]; we did, however, adjust variances for clustering-effects using design-based regression with Taylor linearization method. All analyses were conducted using Stata v16.0 [35]. We followed the STROBE cohort checklist when writing our paper [36].

**Table 1:** Sample characteristics of current hormonal contraceptive or IUD users at baseline, weighted (n = 560)

Variables	Followed-up sample		
	N = 560		
	%	95% CI	
		LL	UL
Age			
Mean (in years)	30.3	29.5	31.2
Married or in-union	86.8	82.1	90.4
Urban	16.7	10.1	26.2
Parity			
0–1	13.9	10.1	18.0
2–3	32.0	23.1	42.5
4+	52.7	45.6	59.8
Education			
None	7.2	4.6	11.0
Primary	57.2	50.5	63.5
Secondary+	35.7	29.1	42.8
Wealth			
Lowest 40%	34.7	28.2	41.8
Highest 60%	65.3	58.3	71.7
Fertility desires			
Want in 1–2 years	12.2	9.1	16.2
Want in 2+ years	42.6	36.8	48.6
Do not want anymore	45.2	39.5	51.1
Using < 12 months	51.4	45.8	56.9
Method			
Implant	31.9	26.8	37.4
IUD	4.6	2.7	7.6
Injectable	56.8	50.6	62.8
Pill	6.8	4.6	9.8
Number of side effects			
None	77.2	72.1	81.7
1	4.8	3.0	7.5
2 or more	18.0	13.8	23.0
Specific side effect			
Less bleeding	9.3	6.2	13.6
More bleeding	5.6	3.5	9.0
Irregular bleeding	7.3	4.4	11.9
Changes to sexual experience	5.4	3.1	9.2
Physical discomfort	13.1	9.2	18.4

**Table 2:** Contraceptive discontinuation, switching, and continuation by method used at baseline, row percentage (weighted)

	Discontinued	Switched	Continued	Total	Total
	%	%	%	%	N
Implants	26.0	12.7	61.2	100.0	187
IUD	15.9	19.4	64.7	100.0	28
Injectables	36.4	24.1	39.5	100.0	300
Pill	32.9	24.4	42.7	100.0	45
Total	30.4	20.6	49.0	100.0	560

**Table 3:** Discontinuation, switching, and continuation by side-effect reported at baseline among current users of hormonal contraception or IUD, row percentage (weighted)

	Discontinued	Switched	Continued	
	%	%	%	N
Any side effect	38.3	23.3	38.4	121
No side-effects	30.1	19.4	50.6	439
Menstrual changes				
Less bleeding	31.8	19.6	31.2	47
More bleeding	41.4	22.8	35.8	30
Irregular bleeding	48.9	17.3	33.9	37
Other bodily changes				
Changes to sexual experience	36.6	22.4	41.1	26
Physical discomfort	32.3	20.6	44.0	70

Women could report multiple side-effects (multiple-response)

## Results

The characteristics of women in the analytic sample are shown in Table 1. The mean age of women in the sample was approximately 30 years old. The majority of women (86.8%) were married or in-union. Less than 15% of contracepting women at baseline were nulliparous or primiparous and the vast majority (approximately 93%) had attended at least some schooling, with the majority attending only primary school (57.2%). Two in three women included in the analytic sample resided in wealthier households. The majority of women wanted to either wait more than two years to their next birth (42.6%) or have no more children (45.2%). About half of women reported that they had been using the current contraceptive method for fewer than 12 months. The majority of women were using the injectable (56.8%) followed by the implant (31.9%).

Approximately one in four of women reported experiencing any side-effects at baseline, while 4.8% were experiencing one side-effect and 18.0% reported experiencing two or more. Physical discomfort was the most commonly reported side-effect reported (13.1%), followed by less bleeding (9.3%).

Table 2 shows the percentage of women who continued the same method at follow-up interview, switched, or discontinued by method type. In total, 47.8% of users were using the same method at follow-up; 20.3% had switched methods; and 31.9% had discontinued use. Switching was highest among pill users, while discontinuation was highest among injectable users.

Table 3 shows the percentage of women who discontinued, switched, and continued by side-effect reported at baseline. Discontinuation was highest among women who reported irregular bleeding (48.9%) and switching was highest among women who reported bleeding more (22.8%) or experiencing changes to sexual experience (22.4%). Continuation was highest among women who reported no side effects.

The adjusted odds ratios for discontinuation and switching relative to continuation are shown in Table 4. Reporting less or more bleeding at baseline increased the odds of discontinuation or switching by 1.86 times (95% CI 1.04–3.34) and 2.74 (95% CI 1.04–7.78) times, respectively. When included in the model alone, irregular bleeding increased the odds of discontinuation and switching by 2.04 times (data not shown: unadjusted OR: 2.04; 95% CI 1.05–3.96, p-value = 0.035), but was not statistically significant in the adjusted model (adjusted OR: 1.31; 95% CI 0.58–2.91). Reporting vaginal dryness/lowered libido and physical discomfort were not associated with discontinuation and switching. Women who used their method for fewer than 12 months had 1.47 times higher odds of discontinuing or switching (OR: 1.47; 95% CI 1.02–2.11), while users of injectables and pills had significantly higher odds of discontinuing or switching than implant users.

**Table 4:** Adjusted odds of discontinuing and switching relative to continuation after adjusting for relevant background characteristics (n = 560)

Variables	Odds ratio (OR)	95% conf. interval		p-value
		LL	UL	
Bleeding less	1.86	1.04	3.34	0.037
Bleeding more	2.74	1.00	7.51	0.050
Bleeding irregularly	1.31	0.58	2.91	0.512
Change in sexual experience	0.95	0.40	2.25	0.913
Physical discomfort	1.24	0.69	2.24	0.470
Using the method < 12 months	1.47	1.02	2.11	0.040
Method (ref: Implant)				
IUD	0.85	0.33	2.17	0.730
Injectables	2.18	1.47	3.23	< 0.001
Pill	3.32	1.68	6.56	0.001
Age	1.02	0.98	1.06	0.261
Parity (ref: 0–1)				
2–3	0.96	0.56	1.64	0.874
4+	0.90	0.45	1.80	0.760
Married or in union (ref: Not married)	0.67	0.41	1.09	0.103
Urban (ref: Rural)	1.34	0.80	2.24	0.261
Education (ref: None)				
Primary	1.01	0.52	1.97	0.976
Higher	1.10	0.51	2.34	0.811
Wealth (ref: lowest 40%)	0.65	0.44	0.96	0.030
Fertility desire (ref: Want 1–2 years)				
Want in 2+ years	0.45	0.25	0.79	0.006
Do not want anymore	0.35	0.18	0.70	0.003

Wealth was significantly associated with discontinuation; women who lived in the wealthiest 60% of housholds had 35% lower odds of discontinuing or switching, relative to continuing (OR: 0.65; 95% CI 0.44–0.96). Women using the method for less than 12 months at baseline had approximately 50% higher odds of discontnuation and switching (OR: 1.47; 95% CI 1.02–2.11). Women who wanted another child in more than 2 years or who did not want another child were significantly less likely to discontinue or switch methods than women who wanted another in 1–2 years [(OR: 0.45; 95% CI 0.25–0.79) and (OR: 0.35; 95% CI 0.18–0.70), respectively].

Table 5 shows the odds ratios of discontinuation compared to switching after adjusting for relevant background characteristics. Overall, there was no difference in the odds of discontinuation compared to switching by reported side-effect. The odds of discontinuing relative to switching decreased with increasing education (p-values < 0.05).

**Table 5:** Adjusted odds ratios of discontinuation relative to switching after adjusting for relevant background characteristics (n = 292)

	Odds ratio	95% conf. interval		P-value
		LL	UL	
Bleeding less	1.30	0.51	3.29	0.581
Bleeding more	1.20	0.38	3.81	0.752
Bleeding irregularly	1.38	0.54	3.49	0.494
Change in sexual experience	0.69	0.21	2.27	0.541
Physical discomfort	0.90	0.37	2.17	0.812
Using the method < 12 months	0.73	0.43	1.25	0.25
Method (ref: Implant)				
IUD	0.45	0.13	1.55	0.203
Injectables	0.98	0.57	1.68	0.944
Pill	0.68	0.29	1.60	0.376
Short-acting method	1.04	0.63	1.70	0.885
Age	0.99	0.94	1.05	0.835
Parity (ref: 0–1)				
2–3	0.63	0.27	1.49	0.289
4+	0.57	0.20	1.60	0.281
Married or in union (ref: Not married)	0.93	0.45	1.93	0.837
Urban (ref: Residence)	1.08	0.67	1.76	0.743
Education (ref: None)				
Primary	0.24	0.07	0.82	0.024
Higher	0.16	0.05	0.54	0.004
Wealth (ref: lowest 40%)	0.75	0.45	1.23	0.247
Fertility desire (ref: Want 1–2 years)				
Want in 2+ years	0.71	0.35	1.44	0.337
Do not want anymore	0.96	0.39	2.36	0.932

## Discussion

We found that approximately one-quarter of women using a hormonal method or the IUD reported currently experiencing at least one side-effect, and that the associations between the experience of side-effects and switching or discontinuation varied substantially based on the nature of the side-effect. Experiencing more or less bleeding increased method specific discontinuation. None of the side-effects examined affected whether women switched versus discontinued, however. These results underscore the importance of examining the unique influence of specific side-effects, instead of the broad singular category of “side-effects and health concerns.” Failure to investigate specific contraceptive side-effects inhibits understanding of nuanced relationships between women’s experiences, their adaptive responses, and subsequent contraceptive behaviors.

Our finding that increased menstrual bleeding was associated with discontinuation aligns with a growing body of literature, highlighting the importance of contraceptive-induced bleeding changes [1, 15, 17]. Excessive bleeding arose as a major barrier to contraceptive use in previous qualitative research in Uganda [12, 37, 38]. One explanation may be that experience of excessive bleeding may present practical challenges to menstrual hygiene management. While limited, research in Uganda has highlighted a range of barriers to effective menstrual hygiene management, resulting from resource limitations and sociocultural norms [39–42]. Despite the conceptual link between contraceptive-induced bleeding changes and menstrual hygiene management, there is extremely limited research available on this subject, and the majority of studies focus on the experience of adolescent girls [42]. Experience of changes in menstrual bleeding may be additionally problematic in environments like Uganda, where myths and misperceptions around the harmful impacts of contraception, including effects on future fertility and on overall health, are widespread [12, 43, 44]. In high-fertility countries such as Uganda, where childbearing is highly valued, changes to menstrual bleeding may not be viewed simply as an inconvenience, but as a prequel to infertility [6, 12]. This belief carries profound social consequences and, therefore, may greatly reduce motivation to use contraception [6, 45].

Evidence from qualitative studies in Africa suggests that amenorrhea and irregular bleeding are often viewed negatively and associated with concerns about future fertility, particularly among nulliparous women [15]. We find that bleeding less increased discontinuation and switching. Similarly, irregular bleeding increased the odds of discontinuation and switching, but was not statistically significant once we accounted for more or less bleeding. Changes to bleeding patterns may affect women’s ability to participate in daily activities, such as religious and work events [10], and the ability to accommodate irregular bleeding is likely to vary across social groups or according to beliefs about potential consequences of such side-effects. Of note, we did not distinguish between less bleeding and cessation of bleeding. Future research should attempt to distinguish between these two bleeding patterns, as complete cessation may engender different behavioral responses than a lighter, but consistent, menstrual period.

We find that vaginal dryness/lowered libido and physical discomfort were not related to an increased odds of discontinuation or switching. Very little research has explored either quantitatively or qualitatively how changes to sexual experience induced by contraceptive use affect contraceptive dynamics, despite evidence that this is an important consideration for both the woman and her partner [10, 12, 16, 46]. Similarly, physical discomfort, which here included headache, nausea, weakness, and cramping, have generally not been included in studies that assess the impact of side-effects. A recent study by Odwe and colleagues found that 30–50% of current and recent injectable and implant users experienced some form of non-bleeding side-effects, often in conjunction with bleeding changes, and that satisfaction with their method was associated with the experience of both bleeding and non-bleeding side-effects [17]. Barden-O’Fallon and colleagues found that abdominal cramping was not associated with the decision to switch versus stop method use. However, the authors did not assess whether cramping increased the overall risk of either relative to continuation [31]. This limits our ability to compare our findings to other studies and underscores the critical need to explore these areas of contraceptive use and experience more fully. We also note that we did not assess severity or frequency of side-effects; women who experience more severe side-effects or more frequent side-effects may discontinue at higher rates than others as Jain and colleagues found in India, adding further nuance to understanding the effect of side-effects on contraceptive behavior [26].

A secondary objective of this paper was to gain a better understanding of the characteristics of women who continue, discontinue, and switch methods after experiencing side-effects, in order to explore how life circumstances influence contraceptive decision-making. Due to sample size limitations, we could not limit our analyses to only women who reported experiencing a side-effect, however, we were able to identify several relevant socio-demographic characteristics associated with discontinuation and switching, after adjusting for the experience of specific side-effects. Wealth was associated with decreased relative risk of discontinuation and switching relative to continuation, while education was associated with an increased risk of switching relative to discontinuation, indicating that social privilege may influence contraceptive dynamics in different ways. For example, social disadvantages that inhibit poorer women from accessing healthcare services to adopt new methods may result in higher discontinuation among this population. Educated women may be more knowledgeable about a range of contraceptive methods than less educated women, allowing them greater opportunity to switch methods if they are dissatisfied. More research on what influences women to discontinue versus switch contraceptive methods after experiencing side-effects is necessary so that family planning programs can better tailor services.

A potential limitation of our study is that women were asked to spontaneously report side-effects that they were currently experiencing, rather than replying directly to a list of potential side-effects. To do so, women needed to consider what they were experiencing as a side-effect of their contraceptive method and, in the case of amenorrhea and loss of libido, recognize the absence or reduction of these experiences as being currently experienced. It is possible that women are less likely to report something that is not happening as being “currently experienced,” relative to something that has increased or newly presented, but this requires further investigation. Additionally, women may not report currently experiencing more bleeding if they are not menstruating. Reading a list of potential side-effects and requiring a response may be a more effective way to gather information on prevalence of side-effects, but may also result in over-reporting [47, 48]. This calls for further methodological research to identify how best to accurately identify and quantify the experience of side-effects in large-scale, population-based surveys.

Under-reporting, in addition to our lower than expected retention, may have contributed to our limited analytical sample size. The limited absolute numbers of women, particularly for switching, restricted our ability to explore relationships across the full range of side-effects. Additionally, our retention at study level was 67%. The application of propensity scores to adjust for loss-to-follow-up allowed us to construct weights that generated estimates of sample characteristics that did not differ between the full baseline and the sample of women who successfully completed follow-up. While we believe we thus adjusted for the majority of demographic and economic characteristics that may be related to contraceptive continuation, there were differences between women who were lost-to-follow-up and those who were included in our sample. Women who were included were older, had higher parity, and were more likely to say they wanted no more children. These women may thus have stronger preferences to prevent childbearing and be more motivated to use contraception [49] despite experiencing side-effects, resulting in conservative estimates of the effect of side-effects; however, as noted, there is little research on tolerability to which we can effectively compare. Finally, we defined switching and discontinuation only based on baseline and follow-up and were unable to account for events that occurred between the two time points, including both the onset of side-effects and switching or discontinuation in the interim. Though we included a contraceptive calendar for this purpose, we found that recall of starting and ending dates of methods when reported retrospectively was inconsistent and rendered the calendar data unusable for this purpose. As we were unable to account for the month-by-month changes between the two surveys, we could have missed behaviors (additional discontinuation/switching of methods) that occurred between the two time points. We believe that this means our estimates are likely conservative, i.e. that we are under-estimating discontinuation and switching and over-estimating continuation.

Despite these limitations, our study has several important strengths. First, we were able to identify and assess the effects of a range of side-effects, including some which have not been extensively studied in the literature. Additionally, we used longitudinal data, following up with women 1 year after the baseline survey, enabling us to assess how these specific side-effects related to discontinuation and switching independently, providing additional insight into contraceptive behaviors.

## Conclusion

Women do not experience generalized “side-effects and health concerns”; they experience specific side-effects and contraceptive behavior is influenced based on both the side-effect and on the woman’s unique circumstances and context. Identifying which side-effects are commonly experienced and strongly associated with discontinuation and switching, in addition to identifying some of the socio-demographic characteristics that may be associated with these behaviors, has valuable programmatic purposes. Counseling that incorporates discussions about individualized suitability for specific side-effects and more discussion about the possibility of switching methods when side-effects do occur may improve continuation rates. Additional research should explore in greater detail the role that context, including partner support, plays in the decision to stop or switch methods.

## Data Availability

The datasets supporting the conclusions of this article are available at www.pmadata.org. All data are available upon request.

## Appendix

See (Tables 6 and 7).

**Table 6:** Sociodemographic characteristics of hormonal contraceptive and IUD users by follow-up status

Variables	Followed-up sample			Lost-to-follow-up sample		
	N = 560			N = 233		
	%	95% CI		%	95% CI	
		LL	UL		LL	UL
Age						
15–24	27.0	22.3	32.4	41.6	33.1	50.7
25–34	43.7	38.8	48.7	39.5	30.7	49.1
35+	29.3	25.1	33.9	18.8	13.3	25.9
Mean (in years)	30.3	29.5	31.2	27.2	26.1	28.3
Married or in-union	86.8	82.1	90.4	73.2	62.9	81.4
Urban	16.7	10.1	26.2	27.3	17.1	40.7
Parity						
0–1	13.9	10.1	18.0	32.0	23.1	42.4
2–3	33.4	27.8	39.4	33.6	25.8	42.3
4+	52.7	45.6	59.8	34.4	25.8	44.2
Education						
None	7.2	4.6	11.0	8.5	4.6	15.2
Primary	57.2	50.5	63.5	56.8	46.2	66.8
Secondary+	35.7	29.1	42.8	34.7	24.7	46.3
Wealth						
Lowest 40%	34.7	28.2	41.8	32.1	23.8	41.6
Highest 60%	65.3	58.3	71.7	67.9	58.4	76.2
Fertility desires						
Want in 1–2 years	12.2	9.1	16.2	17.3	12.1	24.1
Want in 2+ years	42.6	36.8	48.6	50.7	42.8	58.5
Do not want anymore	45.2	39.5	51.1	32.0	24.8	40.2
Using < 12 months	51.4	45.8	56.9	63.2	55.2	70.6
Method						
Implant	31.9	26.8	37.4	35.3	26.8	44.7
IUD	4.6	2.7	7.6	2.5	1.1	5.6
Injectable	56.8	50.6	62.8	53.2	44.7	61.6
Pill	6.8	4.6	9.8	9.0	4.5	17.5
Number of side effects						
None	77.2	72.1	81.7	73.5	65.1	80.6
1	4.8	3.0	7.5	5.3	2.5	10.8
2 or more	18.0	13.8	23.0	21.2	14.2	30.4
Specific side effect						
Less bleeding	9.3	6.2	13.6	10.8	5.7	19.4
More bleeding	5.6	3.5	9.0	5.0	2.5	9.7
Irregular bleeding	7.3	4.4	11.9	6.8	3.5	12.9
Changes to sexual experience	5.4	3.1	9.2	6.6	3.0	13.8
Physical discomfort	13.1	9.2	18.4	15.4	10.5	22.0

**Table 7:** Complete list of potential side-effect answer choices included in survey

Less bleeding
More bleeding
Irregular bleeding
Spotting
Uterine cramping
More cramping
Weight gain
Weight loss
Facial spotting
Headache
Infection
Nausea
Lowered libido
Vaginal dryness
Weakness
Diarrhea
Delayed return to fertility
Infertility
Method lost inside body
Other

## Abbreviations

PMA2020: Performance Monitoring and Accountability 2020
PMA: Performance Monitoring for Action
DHS: Demographic and Health Survey
MICS: Multiple Indicator Cluster Survey
LMICS: Low- and middle-income countries
TFR: Total fertility rate
EA: Enumeration area
IUD: Intrauterine device
OR: Odds ratio
